# Perspectives on Epigenetics and Cancer Immunotherapy: A Preface to Special Issue

**DOI:** 10.3390/cancers13061452

**Published:** 2021-03-22

**Authors:** Damiana Alvarez-Errico

**Affiliations:** Josep Carreras Leukemia Research Institute (IJC), 08916 Badalona, Barcelona, Spain; dalvarez@carrerasresearch.org

The interaction of tumor cells with immune cells within the tumor microenvironment (TME) is the basis for several strategies of tumor evasion from immune surveillance, which nurture cancer development. Epigenetic changes are reversible, heritable covalent modifications that affect gene expression without modifying the DNA’s primary structure, and they include DNA methylation, modification of histone tails, and noncoding (nc) RNAs. Epigenetic alterations drive aberrant expression of tumor-associated genes promoting cellular malignant transformation and cancer progression [1].

Extensive epigenetic reprogramming of immune cells in response to an immunosuppressive TME underlies pro-tumorigenic mechanisms such as T cell exhaustion [2], the polarization of macrophages to an anti-inflammatory phenotype [3], or differentiation into myeloid-derived suppressor cells (MDSCs) [4]. Advancements in the understanding of the molecular mechanisms that control anti-tumor immune responses have fostered the impressive success of immunotherapies for cancer management that we have witnessed in the past few years. Cancer immunotherapies are based on the enhancement of the patient’s immune response to fight the disease and encompass treatment with monoclonal antibodies, immune checkpoint inhibitors, vaccines, or adaptive cell-based therapies [5]. However, despite impressive results for a variety of cancers, the inability of certain individuals to reach full responses and relapse, or the appearance of treatment related toxicities, still preclude many patients from fully benefitting from immunotherapy [6]. Among the regulatory mechanisms that are set in place within the tumor, there is a vast reconfiguration of both the cancer cells and the TME epigenomes that drive a profound reshaping of the epigenetic landscape of tumor-associated immune cells, dampening the anti-tumor response. For instance, the epigenetic repression of immune signature genes that promote effective anti-tumoral responses, such as IFN-γ-mediated Th1 type responses, promotes cancer progression and conditions beneficial clinical responses to immunotherapy [7]. Thus, regarding the epigenetic modulation of tumor-immune cell interactions, epigenetic changes bear a great potential that can be leveraged on two fronts: (i) epigenetic drugs are used to sensitize and potentiate immunotherapeutic responses [8], and (ii) epigenetic modifications can be used as sensitive predictors of the cancer response to immunotherapy [9].

Regarding the combinatorial use of epigenetic drugs with immunotherapy, data show that epigenetic changes observed by treatment with demethylating agents, lead to viral mimicry due to the accumulation of dsRNA on the cytoplasm of cancer cells, triggering INF-type pathways, which promote a strong anti-tumoral response, potentiating immune checkpoint therapy [10].

In this respect, combined treatments that harness the immunomodulatory effect of epigenetic targeting with the antitumor effect of immunotherapy are being explored in clinical trials, with promising results in solid tumors and hematological malignancies [11,12].

Finally, in order to maximize therapeutic success chances, it is key to rely on good predictors of therapy efficacy and/or toxicity. The hypermethylated status of PDCD1LG1 (coding for PD-L1) promoter in patients with recurrent Non-small cell lung cancer (NSCLC) drives the resistance to the anti-PD-1 mAb nivolumab, and its assessment provides valuable information for rational therapeutic decision-making [13]. Beyond the interrogation of the epigenomic status of individual loci, epigenetic profiling may also provide useful markers for resistance/response to immunotherapy. The discovery of a tumor DNA methylation signature named EPIMMUNE revealed that positiveness for such a signature was associated with improved progression-free survival in stage IV NSCLC treated with the anti-PD-1 drugs nivolumab or pembrolizumab [14].

The characteristic of reversibility and sensitivity to changes in the environment of epigenetic modifications make them excellent candidates for biomarkers that may reflect not only individual differences, but also dynamic changes caused by the disease course itself and/or by the treatment.

To summarize, understanding the epigenetic mechanisms underlying the anti-tumor immune response and evasion through cutting-edge research will contribute to the unveiling of potentially targetable immune response boosters through epigenetic drugs and immunotherapy combinatorial approaches, and to the identification of biomarkers of response and resistance to immunotherapies, with the long-term goal of maximizing the clinical benefit of immunotherapies in cancer patients by providing biomarkers for patient selection, allowing for the expansion of the responder population, paving the way for personalized precision medicine.

## References

[1] Feinberg A.P., Vogelstein B. (1983). Hypomethylation distinguishes genes of some human cancers from their normal counterparts. Nat. Cell Biol..

[2] Philip M., Fairchild L., Sun L., Horste E.L., Camara S., Shakiba M., Scott A.C., Viale A., Lauer P., Merghoub T. (2017). Chromatin states define tumour-specific T cell dysfunction and reprogramming. Nature.

[3] Travers M., Brown S.M., Dunworth M., Holbert C.E., Wiehagen K.R., Bachman K.E., Foley J.R., Stone M.L., Baylin S.B., Casero R.A. (2019). DFMO and 5-Azacytidine Increase M1 Macrophages in the Tumor Microenvironment of Murine Ovarian Cancer. Cancer Res..

[4] Huang S., Wang Z., Zhou J., Huang J., Zhou L., Luo J., Wan Y.Y., Long H., Zhu B. (2019). EZH2 Inhibitor GSK126 Suppresses Antitumor Immunity by Driving Production of Myeloid-Derived Suppressor Cells. Cancer Res..

[5] Villanueva L., Álvarez-Errico D., Esteller M. (2020). The Contribution of Epigenetics to Cancer Immunotherapy. Trends Immunol..

[6] Kennedy L.B., Salama A.K.S. (2020). A review of cancer immunotherapy toxicity. CA A Cancer J. Clin..

[7] Peng D., Kryczek I., Nagarsheth N., Zhao L., Wei S., Wang W., Sun Y., Zhao E., Vatan L., Szeliga W. (2015). Epigenetic silencing of TH1-type chemokines shapes tumour immunity and immunotherapy. Nat. Cell Biol..

[8] Topper M.J., Vaz M., Marrone K.A., Brahmer J.R., Baylin S.B. (2020). The emerging role of epigenetic therapeutics in immuno-oncology. Nat. Rev. Clin. Oncol..

[9] Sundar R., Huang K., Qamra A., Kim K.-M., Kim S., Kang W., Tan A., Lee J., Tan P. (2019). Epigenomic promoter alterations predict for benefit from immune checkpoint inhibition in metastatic gastric cancer. Ann. Oncol..

[10] Chiappinelli K.B., Strissel P.L., Desrichard A., Li H., Henke C., Akman B., Hein A., Rote N.S., Cope L.M., Snyder A. (2015). Inhibiting DNA Methylation Causes an Interferon Response in Cancer via dsRNA Including Endogenous Retroviruses. Cell.

[11] Di Giacomo A.M., Covre A., Finotello F., Rieder D., Danielli R., Sigalotti L., Giannarelli D., Petiprez F., Lacroix L., Valente M. (2019). Guadecitabine plus ipilimumab in unresectable melanoma: the NIBIT-M4 clinical trial. Clin. Cancer Res..

[12] Daver N., Garcia-Manero G., Basu S., Boddu P.C., Alfayez M., Cortes J.E., Konopleva M., Ravandi-Kashani F., Jabbour E., Kadia T.M. (2019). Efficacy, Safety, and Biomarkers of Response to Azacitidine and Nivolumab in Relapsed/Refractory Acute Myeloid Leukemia: A Nonrandomized, Open-Label, Phase II Study. Cancer Discov..

[13] Zhang Y., Xiang C., Wang Y., Duan Y., Liu C., Zhang Y. (2017). PD-L1 promoter methylation mediates the resistance response to anti-PD-1 therapy in NSCLC patients with EGFR-TKI resistance. Oncotarget.

[14] Duruisseaux M., Martínez-Cardús A., E Calleja-Cervantes M., Moran S., de Moura M.C., Davalos V., Piñeyro D., Sanchez-Cespedes M., Girard N., Brevet M. (2018). Epigenetic prediction of response to anti-PD-1 treatment in non-small-cell lung cancer: a multicentre, retrospective analysis. Lancet Respir. Med..

